# Confirming the Efficacy of an Adaptive Component to Family-Based Treatment for Adolescent Anorexia Nervosa: Study Protocol for a Randomized Controlled Trial

**DOI:** 10.3389/fpsyt.2020.00041

**Published:** 2020-02-12

**Authors:** Alexa L’Insalata, Claire Trainor, Cara Bohon, Sangeeta Mondal, Daniel Le Grange, James Lock

**Affiliations:** ^1^ Department of Psychiatry and Behavioral Sciences, Stanford University School of Medicine, Stanford, CA, United States; ^2^ Department of Psychiatry, University of California, San Francisco, San Francisco, CA, United States; ^3^ Department of Psychiatry and Behavioral Neuroscience, The University of Chicago, Chicago, IL, United States

**Keywords:** anorexia nervosa, eating disorder, family based treatment, adolescents, randomized controlled trial

## Abstract

Family-based treatment (FBT) has the largest evidence base for treating adolescents with anorexia nervosa (AN); 35–50% of cases remit at the end-of-treatment and remain remitted 3–4 years after treatment. Studies of FBT demonstrate that weight restoration by session 4 (of 2.4 kgs) predicts remission at end of treatment in 85–90% of cases. One way to improve outcomes is to tailor treatments to patients depending on successful weight restoration at session 4. Pilot data found that by adding three sessions of Intensive Parental Coaching (IPC) after session 4 improved outcomes in early non-responders. Further, data suggest that the mechanism underlying FBT is early improvements in parental self-efficacy related to re-feeding their child. This manuscript describes a study protocol to examine whether adding IPC to FBT improves outcomes in early non-responders and confirm whether change in parental self-efficacy is the mechanism by which FBT works. This two-site randomized controlled trial (RCT) will randomize 60 adolescents with a DSM-5 diagnosis of AN (30 per site) who are between the ages of 12–18 years old and do not gain 2.4 kgs by session 4 of FBT. Randomized participants will either continue standard FBT or receive the three sessions of IPC and then continue FBT as usual. Both arms include up to 18 sessions over the course of 9 months. Blinded assessments will be conducted at baseline, 3-month within-treatment, end of treatment, and at 6 and 12-month follow-up. Parental-self efficacy will be assessed using the Parent versus Anorexia Scale at all major assessment time points and at each of the first eight sessions of treatment. The primary outcome is achievement of weight remission (> 94% expected mean percent body mass index (BMI) adjusted for age, height, and gender).

**Clinical Trial Registration:**

http://www.ClinicalTrials.gov, identifier NCT03097874.

## Introduction

For adolescents with anorexia nervosa (AN), family-based treatment (FBT) has the largest evidence base of effectiveness. FBT yields better outcomes than adolescent focused therapy (AFT) (1) for adolescents with AN. While there is some evidence supporting the efficacy of enhanced cognitive behavior therapy (CBT-E) for adolescents with AN (2), FBT remains the first-line approach for adolescent AN. Some 35–52% (3, 4) of cases fully recover and remain remitted 3–4 years after treatment (5–7). However, those who do not recover have a high risk of developing enduring AN for which there is no evidence-based treatment (8) which contributes to reduced quality of life and increased risk for premature death (9). Studies of FBT find that weight restoration by session 4 (of 2.4kgs) predicts outcome (e.g. weight restoration at end of treatment) in 85–90% of cases (10–13). Among the patients who do not meet weight restoration targets by session 4 under standard FBT, only 14% recover. Previous studies have also shown that FBT targets parental self-efficacy (SE) at re-feeding as a mechanism to promote weight restoration (14, 15). Families with lower parental SE tend to be less likely to reach weight restoration at end of treatment. According to pilot study data (16), the recovery rate of poor early responders who were treated with only three additional sessions focused on improving parental self-efficacy increased to 58.3%. The aim of this study is to confirm whether adding an adaptive treatment compatible with FBT significantly improves outcomes by addressing poor early response, including targeting parental SE, and to evaluate SE as a mechanism of treatment effect in FBT.

## Study Design

A pilot study investigated the development and implementation of a three-session intensive parental coaching (IPC) for adolescents with AN receiving FBT who had failed to meet weight restoration targets by session 4. Participants receiving IPC showed increased parental skills at re-feeding compared to those who did not receive the adaptation. Overall, results from this study demonstrated the feasibility of using a randomized adaptive design employing IPC combined with FBT. Parents whose children did not gain 2.4 kgs by session 4 reported lower levels of SE related to re-feeding as early as session 2 (16), however, after receiving FBT+IPC, parental SE scores in this group improved and became indistinguishable from those of parents of early responders (16). We hypothesize that low parental SE leads to failure to deploy adequate re-feeding behaviors. Thus, a sufficiently powered and controlled randomized controlled trial (RCT) using an adaptive randomized design and employing IPC as the adaptive intervention for poor early responders was developed to confirm the efficacy of the adaptive intervention and examine mechanisms and moderators of treatment. The study was designed to address the following specific aims:

- Aim 1: To conduct an RCT using an adaptive design for adolescents (ages 12–18 years) with DSM-5 AN comparing standard FBT to adaptive FBT (FBT+IPC).
- Aim 2: To examine parental SE related to re-feeding as a mediator of treatment effect (standard vs. adaptive) on expected body weight (EBW) at end of treatment (EOT). We will also explore the potential mediating effects of weight restoration and changes in parental re-feeding behavior.
- Aim 3: To evaluate target engagement of parental SE within adaptive FBT and to explore target engagement of parental re-feeding behaviors within adaptive FBT.
- Aim 4: To examine moderators of treatment outcome. We will explore all baseline variables as potential predictors and/or moderators of outcome.

## Participants

We plan to initially recruit approximately 150 adolescents, 75 at each of two sites (Stanford University and the University of California, San Francisco). Participants who have gained 2.4 kgs by session 4 will continue to complete up to 18 sessions of standard FBT over 9 months. Participants who do not meet weight restoration targets will be randomized to either receive IPC for three sessions starting at session 4 and then continue to complete standard FBT or randomized to complete standard FBT as usual (total of up to 18 sessions over 9 months in both treatments). Of the initial approximately 150 participants recruited, we expect 30–40 per site to be randomized (for a total of 60–75 randomized adolescents). This rate of randomized participants will allow for an adequately powered study (power = 0.87, alpha = .05, two-tailed) to address our primary aim. An attrition rate was predicted as part of the initial power calculations.

In addition, of those participants randomized, 20 randomly selected participants receiving FBT+IPC using coded mealtime behaviors from session 2 (coached meal 1 in FBT) will be compared to those in session six (coached meal 2 in FBT+IPC) using observational data gathered from coded videotapes following established behavior coding frames (17). We predict there will be a significant increase in re-feeding behaviors consistent with successful re-feeding at meal 2 (session six) compared to meal 1 (session 2) among these randomly selected participants.

### Inclusion/Exclusion/Withdrawal Criteria

The inclusion criteria for study participants includes: 1) A child that meets DSM-5 criteria for AN (both subtypes) who is between the ages of 12 and 18 years; 2) Child who has an expected mean body weight percentage (%EBW) >75.0% and <88.0%; 3) Medically stable for outpatient treatment according to the recommended thresholds of the American Academy of Pediatrics and Society of Adolescent Medicine (18); 4) Child not engaging in another individual or family based psychotherapy trial during the duration of treatment sessions with the study; 5) Child and parents who speak and read English fluently.

Potential participants will be excluded for any of the following reasons: 1) Current psychotic illness, mental retardation, and/or other mental illnesses that would prohibit the use of psychotherapy; 2) Current dependence on drugs or alcohol; 3) Physical conditions (e.g. diabetes mellitus, pregnancy) known to influence eating or weight; 4) Previous FBT (four or more sessions); 5) Currently taking adjunctive medication for co-morbid disorders (e.g., antidepressants) for less than 2 months (8 weeks) that cannot be safely discontinued prior to treatment; 6) In the case of patients with current, or a history of sexual or physical abuse by family members, perpetrators of the abuse will be excluded from treatment.

Participants will be withdrawn from the study if any of the following occur while in treatment: 1) Sexual or physical abuse by a family member (perpetrators will be withdrawn; child may stay in treatment) 2) Hospitalization for >21 consecutive days; 3) Missing >4 consecutive scheduled appointments; 4) Worsening of psychiatric conditions such that participant would be clinically better served by referral for other treatment; 5) Participant undertaking other psychotherapies during the treatment study.

## Collaborating Sites

The treatment sites are directed by established clinicians and researchers in the field of eating disorders with expertise in FBT. Both sites have extensive experience and success recruiting adolescents for RCTs for the treatment of eating disorders and both have trained personnel in FBT and IPC who participated in the feasibility pilot study. We will minimize the site effect by following the manualized protocol and using highly trained therapists. Nonetheless, we expect some treatment effect heterogeneity across sites as the study participants will be recruited from the diverse population surrounding UCSF and Stanford. We will closely monitor both the sites and site by treatment interaction effects, and our analyses will be conducted allowing for differences across the two sites.

## Recruitment

Families in the San Francisco Bay Area with a child with a diagnosis of Anorexia Nervosa will be recruited through colleague referrals, community organizations, clinics treating eating disorders, social media (i.e., Twitter, Facebook), and by publicizing in the local media. A population-based estimate (1% of adolescent females in the tri-county referral area for our clinics) would predict that approximately 2,300 of 230,000 adolescent females in the area would meet AN DSM-5 diagnostic criteria. During the calendar year 2013–2014, both sites had over 300 referrals to their respective adolescent eating disorder programs that would meet inclusion criteria and be eligible for recruitment. Any family that calls or emails expressing interest will be contacted by the research staff to describe the study and screened over the phone for eligibility. Research staff will provide information about study procedures, including randomization, over the phone and answer any questions families may have about the study. Of note, study hypotheses about treatment arms are not included in this description. Families are told that the effectiveness of IPC is unknown and that we hope to better identify an adequate treatment for early non-responders through these trials. If participants meet initial criteria *via* phone screen, they will be asked to schedule and attend a baseline assessment if they wish to continue with the study. During the baseline assessment, trained research coordinators will obtain informed consent and assess the target participant to further determine eligibility. If families are still eligible and interested after the baseline assessment, they will be scheduled to start treatment with an FBT certified provider trained in IPC.

## Assessment

The inclusion and exclusion criteria for the child will be assessed at the baseline assessment. Inclusion criteria will be determined by calculating expected mean body weight percentage (%EBW) from height and gowned weight collected, self-report assessments by parent and child, the eating disorders examination (19), and study physician clearance after baseline. Additionally, child psychopathology assessed *via* the Kiddie Schedule for Affective Disorders and Schizophrenia (KSADS) (20) will be evaluated at baseline for secondary data analysis (i.e. comorbidity) and for exclusion criteria (i.e., psychosis). The eating disorder examination (EDE) and %EBW will also be collected at every other assessment time point (3-month within-treatment, end of treatment, 6- and 12-months post treatment) as outcome measures. Assessments and measures along with the time points that they will be administered are listed in **Tables 1** and **2**.

**Table 1:** Patient Self-report measures.

Measure	BL	Session	3- month	EOT	6-month Follow-up (15 months)	12-month Follow-up (21 months)
TSPE		End of session 1				
HRQ		End of session 1	x			
Weight (Primary outcome; from EDE)	x	Session 4	x	x	x	x
Weight by therapist (HW Log)		Every session				
EDE	x		x	x	x	x
YBC-ED	x		x	x	x	x
BDI	x	1 - 8	x	x	x	x
BAI	x	1 - 8	x	x	x	x
CY-BOCS	x		x	x	x	x
CET	x	Session 4				
CES	x	Session 4				

**Table 2:** Parent Self-report measures.

Measure	BL	Session	3- month	EOT	6-month Follow-up (15 months)	12-month Follow-up (21 months)
TSPE		End of session 1				
HRQ		End of session 1	x			
PvAN	x	1 - 8	x	x	x	x
SDQ	x	1 - 8	x	x	x	x

### Primary Measures

#### Percent Expected Body Weight (%EBW)

Will be calculated using a computer program based on the Center for Disease Control Growth Charts with weight adjusted for age, gender, and height. This formula for calculating %EBW has been used in the previous RCTs of adolescents with AN (16). Achievement of >94% EBW will be the primary outcome. The assessor will obtain the potential participant's height and weight at baseline and follow up assessment time points. Weight will be assessed by means of a balance beam scale, calibrated daily, with participants in a hospital gown and no shoes. Height will be assessed using a stadiometer, calibrated in 1/8inch intervals. %EBW will be calculated from height (in meters) and weight (in kilograms) data. Height and weight along with %EBW calculation will occur at all major assessment points.

#### Target Engagement

Change in parental self-efficacy as a mechanism of therapeutic effect will be examined with the *Parents versus Anorexia Nervosa Scale (PvAN)* (21). It is a self-efficacy measure designed after Bandura's general self-efficacy measure, specifically focused on parental self-efficacy related to re-feeding their son or daughter with AN. The measure has been used in multiple studies and has good internal consistency. Parent participants in the study will complete this questionnaire at baseline, weeks 1–8 and all other major assessment points.

### Secondary Measures

#### Eating Disorder Examination (EDE)

A standardized investigator-based interview assessing the severity of the characteristic psychopathology of eating disorders. The psychometric properties of the EDE are sound and have been used in many treatment studies. Version 16 of the assessment will be used by trained research coordinators who will be administering this assessment to adolescent participants at all major assessment points (19).

#### Yale-Brown-Cornell Eating Disorder Scale (YBC-ED)

The YBC-ED is a clinician rated scale assessing impairment, persistence and degree of obsessional thinking and compulsiveness about eating thoughts and behaviors (22). Again, research coordinators will administer this assessment at all major assessment points.

#### Beck Depression Inventory (BDI)

The BDI is self-report measure assessing for depressive symptoms in adolescents (23). This scale has been used in numerous studies of adolescent depression. This measure also includes questions about suicidal ideation, plan, and intent and will be used to assess suicide during the study. If participants endorse suicidal ideation with a plan or intent, this information will be reported to the site PI and/or treating clinician, who will perform appropriate clinical intervention. Participants will complete this measure weekly for weeks 1–8 and at all major assessment points.

#### Beck Anxiety Inventory (BAI)

The BAI is a 21-item self-report measure assessing symptoms of anxiety (24). Adolescent participants will complete this measure at baseline, sessions 1–8, and all major assessment time points. We will evaluate the feasibility of the BAI for use in future studies to examine mediation.

#### Children's Yale-Brown Obsessive Compulsive Scale (CY-BOCS)

The CY-BOCS (25), a modified version of the Y-BOCS, is a 10-item, clinician-rated, semi-structured instrument assessing symptom severity of Obsessive-Compulsive Disorder (OCD). This measure will be administered at all major assessment points by a trained research coordinator.

#### Therapy Suitability and Patient Expectancy (TSPE):

The TPSE measures perceptions of the suitability and expectancy of the treatment provided. It will be rated by both adolescent and parent participants on a visual analogue scale (0–10) at the end of session 1.

#### Helping Relationship Questionnaire (HRQ)

The HRQ is an 11-item questionnaire used to measure the quality of the therapist-patient relationship across the two treatments (26). The HRQ will be completed by parent participants after the first treatment session and at the 3-month within-treatment assessment time point.

#### Parental Mealtime Behaviors

Treating clinicians will videotape sessions 1 through 8 for randomized non-responders and sessions 1 through 4 for responders. These tapes will include the family meal at session 2 (and, if applicable, session six for patients receiving IPC). For those participants randomly selected for the videotape analyses, parental re-feeding behaviors at family meals will be coded using procedures and coding frames used in preliminary studies (17).

#### Compulsive Exercise Test (CET)

The CET is a 24-item multidimensional measure designed to assess core factors operating in the maintenance of excessive exercise specifically among patients with eating disorders (27). Adolescent target participants will complete this measure at baseline and at session 4.

#### Commitment to Exercise Scale (CES)

Similar to the CET, the CES evaluates the degree of psychological commitment to exercising (and its corresponding attitudinal and behavioral features) (28). This 8-item measure will be completed by the target child at baseline and at session 4.

## Washout Period Prior to Randomization

Approximately 150 adolescents with AN will receive initial FBT (four sessions). Based on the largest study on FBT (29), 44% are categorized as having a poor response by session 4 under standard FBT. Thus, we expect that 40–50% of participants (i.e., 60 to 75 out of 150) will be early non-responders to FBT. Early non-responders would then be randomized within site at session 4 to either FBT or FBT+IPC conditions and repeatedly assessed, allowing for strong inference regarding the effects of FBT+IPC (compared to FBT only) on adolescent AN patients. Participants who are not randomized (responders) at session 4 will still complete follow-up assessments and provide data, but their data will not be a part of primary analyses.

It is expected that participant treatment withdrawal will be minimum as FBT is considered to be a first line treatment for adolescent AN, so feasibility of recruitment is likely further enhanced in this study by the fact that all participants will receive standard FBT at a minimum and at no cost. To help assure maintenance of the randomized participants, research staff and clinicians will emphasize building rapport with the families to increase treatment adherence and prolonged study adherence. Surveys at sessions 1 through 8 will help with building continuous rapport with the families. In addition, to further ensure that families complete follow up assessments at the 6- and 12-month post treatment time points, patients will receive $50 at the completion of the 12-month follow-up assessment.

## Randomization

Prior to the start of session 4, therapists will weigh patients with a research coordinator observing. The research coordinator will calculate weight difference between sessions 1 and 4 using a calculator, calculating weight difference twice to verify results. Patients will be considered treatment responders if weight restoration is greater than or equal to 2.4 kgs. Patients will be considered treatment non-responders if they fail to gain 2.4 kgs. After determining the responder status, the research coordinator will determine if randomization is needed. Responders will not be randomized; therefore, the therapists will be notified to continue with FBT. Prior to the start of the study, the Data Coordinating Center provided each site with 32, sequentially numbered, sealed envelopes. This envelope will be given to the therapist following the weighing at session four informing them and not the study coordinator/assessor of the randomized assignment. This system allows for efficient randomization and serves to keep research coordinators blinded to treatment condition. If a patient is hospitalized for medical instability after session 1, but before session 4, any weight gained in the hospital is subtracted from the total weight difference at session 4 to allow for accurate calculation of *out patient* weight gain during the first 4 sessions of FBT.

## Interventions/Treatments

### Family-Based Treatment (FBT)

FBT is a manualized treatment divided into three phases (30). In the version used in this study, the first phase (sessions 1–8), focused on the eating disorder and included a family meal. This phase is characterized by attempts to absolve the parents from the responsibility of causing the disorder (agnosticism), separating the child from the eating disorder (externalization), and by encouraging positive aspects of their parenting (parental empowerment). In consultation with their therapist, families are encouraged to work out for themselves how best to re-feed their child, which helps to bolster parental empowerment and self-efficacy. In Phase 1, parents are solely responsible for the re-nourishment of their child, including preparing and supervising meals and snacks. In Phase 2 (sessions 8–14), once weight restoration is nearing completion, therapists help parents to transition eating and weight control back to the adolescent in an age appropriate manner. The third phase (sessions 14–18) is initiated after the patient achieves a healthy weight and eating disorder behaviors (i.e. self-starvation, compensatory behaviors) have abated. The central theme in this phase is the establishment of a healthy adolescent or young adult relationship with the parents. Sessions last 1 h and are generally held on a weekly basis during most of phase 1, but are titrated to every 2–4 weeks in later phases depending on progress. A total of 18 sessions are provided over 9 months, the same as the other treatment arm. Use of all 18 sessions is not mandatory to complete treatment, and some families may complete treatment with fewer than 18 sessions or in less than 9 months (30).

### Intensive Parental Coaching (IPC)

The stepped care intervention that will be used is FBT+IPC. This new treatment was developed and piloted, refined, and piloted again in an iterative process involving 21 cases prior to finalizing the format used in a feasibility study (16). In the adaptive treatment arm, FBT+IPC provides three sessions added to standard FBT focused on mealtime coaching for families whose child had not gained 2.4 kgs by session 4. Studies have suggested that direct coaching at mealtimes improves parental efficacy (17). The first of these sessions (IPC session 1) is a family session designed to present the failure to gain sufficient weight by this point in treatment as a crisis and re-invigorate the family to make definitive behavioral changes to support weight restoration. Following this session, a session with parents alone (IPC session 2) is held to identify what impediments the parents perceive might be interfering with successful re-feeding. Finally, a second family meal (IPC session 3) is held, which includes direct coaching by the therapist to help the parents address the specific challenges identified during the meeting with the parents alone. Following these three sessions, the treatment resumes the regular course of standard FBT. Thus, a total of 18 sessions are provided over 9 months, the same as the standard FBT arm. Again, patients are not required to use all 18 sessions, and some may complete treatment in fewer sessions or in less than nine months.

### Therapist Training and Supervision

Each site will have a minimum of two therapists providing treatment through the research study, and all therapists will conduct both treatments. All providers are PhD clinical psychologists with experience in treating adolescents with AN. They will not be involved in research assessments. In addition, a treatment supervisor will be trained at each site. Training will involve the following: Therapists and supervisors will familiarize themselves with the manuals by reading specified preparatory materials (manuals and chapters describing FBT and FBT+IPC); Drs. Lock and Le Grange will conduct a two-day intensive workshop at the start of the study on the treatments which includes role-play and active rehearsal of the treatments. This workshop will be recorded for future reference and training for site therapists. Each therapist will attend weekly supervision by site principal investigators. During supervision each patient's progress is reviewed as well as the therapists' adherence to the treatment protocols.

### Therapeutic Adherence and Treatment Fidelity

We will assess adherence and fidelity to FBT and FBT+IPC by evaluating videotapes of sessions 1–3 (FBT) and 4–6 (IPC) on a random sample of 20% of sessions employing the standardized fidelity measure focused on early FBT treatment (31). Because IPC, like FBT, focuses on enhancing parental self-efficacy through agnosticism, externalization, and focused re-feeding strategies using a second family meal (session 6) we will use this same validated fidelity measure to examine sessions 4–6 of IPC.

### Adequate Dose of Treatment

For those participating in the randomized study an adequate dose of treatment is defined as eight sessions. The rationale for this decision is that this number includes three sessions prior to randomization, the three additional sessions of IPC post-randomization, and two standard FBT sessions following the completion of IPC. Those who do not complete eight sessions of treatment (IPC or continued FBT) will be regarded as treatment dropouts if they are willing to complete all or part of the standard assessments. If they are not willing to complete all or part of the standard assessments following the early termination of treatment they will be regarded as study dropouts.

## Data Collection and Management

All data at both UCSF and Stanford University will be collected by trained and certified assessment staff. All data will be stored by the Data Coordinating Center (DCC) housed at Stanford University on a dedicated server with secure access available to both the statistician and the DCC Data Manager. All data will be checked following participant assessments. All non-web-based data will be scored, entered, and checked by the DCC. Web based data collection will use the Qualtrics survey tool. The DCC Data Manager and statistician will be responsible for developing and maintaining the database including the creation of electronic case report forms (eCRFs) and data pipeline according to study design, performing quality control measures, assisting in protocol development, writing reports, building datasets appropriate for statistical analysis, and submitting data/documents to regulatory bodies (e.g. IRB and NIMH). Statistical analysis will utilize SPSS 25.0 as well as specialized statistical programs when necessary. All software will be updated yearly.

## Statistical Analyses

The primary outcome of our study is remission (weight > 94% EBW) by EOT. The core analysis strategy in this project is mixed effects (ME) modeling (9), where we fully utilize the repeatedly measured primary and secondary outcomes (from major assessment timepoints: baseline, 3-month within-treatment, EOT, 6- and 12-month follow-up). For maximum likelihood (ML) estimation of mixed effects models, the Mplus program version 7.4 or above will be used (32). Data points that are missing due to attrition or missing assessments will be handled assuming that data are missing at random conditional on observed information (1). The results of these analyses can be easily converted to randomized group differences at EOT and follow up assessments. The analysis is Intent to Treat using mixed effects modeling and all participant data will be included (with the exception of PI Withdrawals) in the analysis.

In Aim 1, we will compare the remission rate between the FBT and the adaptive FBT (FBT vs. FBT+IPC) groups at EOT in line with the intention to treat principle. For this comparison, we will conduct mixed effects growth modeling utilizing remission until EOT, but also including the full set of four repeated measures of remission (3-month mid-treatment, EOT, 6- and 12-month follow-up) as there is no variation in the data at baseline (i.e., no one experiences remission yet by design). We will use a random intercept/slope model treating remission status as a binary outcome (weight > 94% EBW = remission; weight < = 94% EBW = not in remission). All individuals who had the remission information at one or more assessment points will be included in our analysis. We will also evaluate %EBW as a secondary outcome using ME models to analyze %EBW as a continuous outcome.

In Aim 2, we will examine whether early change (from baseline to session 8) in parental self-efficacy mediates (i.e., treatment mechanism) the treatment effect (FBT vs. FBT+IPC) on remission at EOT. We will follow the eligibility and analytical criteria of MacArthur approach for mediator analysis. To maximize power to identify the mediator given our moderate sample, actual estimation of mediator effects will be conducted using mixed effects modeling fully utilizing all available longitudinal data. In addition to the longitudinal recovery data (described in Aim 1), we will utilize longitudinally measured mediator (self-efficacy measured at baseline and at sessions 1–8). We will also examine early changes in weight restoration and parental re-feeding behavior as potential mediators using the same analysis strategy.

In Aim 3, we will examine the target engagement by monitoring the change in parental re-feeding behaviors in a subset (N = 20) of randomized participants to FBT+IPC. We will compare parental mealtime behaviors at session 2 (pre-randomization) using behavioral data gathered from coded videotapes to coded mealtime behaviors from videotaped recordings of the second coached meal provided in FBT+IPC (session 6). We will employ pairwise t-test and mixed effects modeling for this within group investigation of behavioral change. We will also explore association between parental self-efficacy and these behavioral changes. For this investigation, using the same subset of subjects (N = 20), we will first examine correlation (Spearman) between self-efficacy and parent behavior at session 2 and session 6. Second, using mixed effects modeling, we will examine how the longitudinal change in self-efficacy (measured at baseline and at Sessions 1–8) is related to the change in parent feeding behavior.

In Aim 4, we will examine baseline moderators of treatment effect on remission. In particular, we hypothesize that family structure and higher levels of eating related obsessions will moderate the treatment effect. For this investigation, we will follow the eligibility and analytical criteria of MacArthur framework for moderator analysis (10, 16, 33).

## Discussion

FBT has the largest evidence base of treatments for adolescents with AN. However, despite remission rates of 30–50%, those who do not respond early with sufficient weight restoration are much less likely to recover. In addition, preliminary data suggest that early response is correlated with greater parental self-efficacy (14, 16). In summary, this multisite study aims to examine whether the efficacy of FBT can be increased for those who fail to respond by robust weight gain (2.4 kgs) by week 4 of treatment by adding additional focused content designed to increase parental self-efficacy. Based on preliminary data, it is hypothesized that increasing parental self-efficacy at re-feeding their child through three additional sessions of intensive parental coaching will lead to a 30% increase in remission rate. The primary outcome variables for this study will be %EBW and EDE scores at end of treatment and 6- and 12-month post treatment assessments and changes in parental self-efficacy as observed through the PvAN (1, 5, 16). Additional outcome variables include depression and anxiety scores, OCD symptoms, eating disorder related obsessionality and compulsivity, and exercise behaviors relating to the eating disorder.

If results from this study show that early non-responders who receive IPC+FBT have better outcomes than those who only receive FBT, this adapted treatment can be disseminated and improve overall remission rates for patients with AN receiving FBT. This is important because clinicians are uncertain about how to assist families where the child does not gain sufficient weight early in FBT. Providing a clear adaptive approach to improve outcome can support these families and prevent an enduring course of illness. Further, results may help to confirm past research showing that parental self-efficacy is the mechanism for how FBT works. Understanding the mechanism underlying the treatment effects of FBT could help identify ways to enhance this mechanism and potentially further improve outcomes.

## Ethics Statement

This protocol involving human participants was reviewed and approved by Stanford University Institutional Review Board (IRB). Written informed consent to participate in this study will be obtained from each participant or the participant's legal guardian.

## Author Contributions

JL and DLG contributed to the scientific specification and conceptualization of the study protocol. AL, CT, CB, and SM contributed to the writing of the study protocol. All authors contributed to the writing of the manuscript.

## Funding

The National Institute of Mental Health is providing financial support for this study through 1R01MH110538-01A1.

## Conflict of Interest

JL receives royalties from Guilford Press and Routledge. He also is co-director of the Training Institute for Child and adolescent Eating Disorders, LLC. DGL also receives royalties from Guilford Press and Routledge. He also is co-director of the Training Institute for Child and adolescent Eating Disorders, LLC.

The remaining authors declare that the research was conducted in the absence of any commercial or financial relationships that could be construed as a potential conflict of interest.
